# The glycosyltransferase EXTL2 promotes proteoglycan deposition and injurious neuroinflammation following demyelination

**DOI:** 10.1186/s12974-020-01895-1

**Published:** 2020-07-23

**Authors:** Annie Pu, Manoj K. Mishra, Yifei Dong, Samira Ghorbanigazar, Erin L. Stephenson, Khalil S. Rawji, Claudia Silva, Hiroshi Kitagawa, Stephen Sawcer, V. Wee Yong

**Affiliations:** 1 Department of Clinical Neurosciences and the Hotchkiss Brain Institute, University of Calgary, T2N 4 N1, Calgary, Canada; 2 Kobe Pharmaceutical University, Kobe, Japan; 3 University of Cambridge, Cambridge, UK; 4 University of Calgary, 3330 Hospital Drive, Calgary, Alberta T3A 4X9 Canada

**Keywords:** CSPG, Multiple sclerosis, Inflammation, Demyelination, Oligodendrocyte, EXTL2

## Abstract

**Background:**

Chondroitin sulfate proteoglycans (CSPGs) are potent inhibitors of axonal regrowth and remyelination. More recently, they have also been highlighted as a modulator of macrophage infiltration into the central nervous system in experimental autoimmune encephalomyelitis, an inflammatory model of multiple sclerosis.

**Methods:**

We interrogated results from single nucleotide polymorphisms (SNPs) lying in or close to genes regulating CSPG metabolism in the summary results from two publicly available systematic studies of multiple sclerosis (MS) genetics. A demyelinating injury model in the spinal cord of exostosin-like 2 deficient  (EXTL2^-/-^) mice was used to investigate the effects of dysregulation of EXTL2 on remyelination. Cell cultures of bone marrow-derived macrophages and primary oligodendrocyte precursor cells and neurons were supplemented with purified CSPGs or conditioned media to assess potential mechanisms of action.

**Results:**

The strongest evidence for genetic association was seen for SNPs mapping to the region containing the glycosyltransferase exostosin-like 2 (EXTL2), an enzyme that normally suppresses CSPG biosynthesis. Six of these SNPs showed genome-wide significant evidence for association in one of the studies with concordant and nominally significant effects in the second study. We then went on to show that a demyelinating injury to the spinal cord of EXTL2^−/−^ mice resulted in excessive deposition of CSPGs in the lesion area. EXTL2^−/−^ mice had exacerbated axonal damage and myelin disruption relative to wild-type mice, and increased representation of microglia/macrophages within lesions. In tissue culture, activated bone marrow-derived macrophages from EXTL2^−/−^ mice overproduce tumor necrosis factor α (TNFα) and matrix metalloproteinases (MMPs).

**Conclusions:**

These results emphasize CSPGs as a prominent modulator of neuroinflammation and they highlight CSPGs accumulating in lesions in promoting axonal injury.

## Background

Multiple sclerosis (MS) is an inflammatory disease of the central nervous system (CNS) with focal areas of demyelination induced by aberrant immune activation. Associated with demyelination and inflammation are a multitude of changes in the composition of the CNS extracellular matrix (ECM) (reviewed in [1, 2]); however, changes in ECM are understudied in the context of MS, where lesional alterations are largely undefined [3]. Previous reports have highlighted the deposition of chondroitin sulfate proteoglycans (CSPGs) at the borders of chronic active MS lesions [4]. CSPGs are a class of large, glycosylated proteins present both on cell surfaces and in the extracellular matrix. Lecticans, a subset of ECM CSPGs, homeostatically comprise a significant portion of perineuronal nets as well as the neural interstitial matrix, serving roles in development, plasticity, and neuronal function [5, 6]. Lectican CSPG members are versican, aggrecan, neurocan, and brevican. In demyelinating injury, they are locally upregulated by several cell types and deposited near the injury site [7–13]. CSPGs are potent inhibitors for regenerative processes such as axonal regeneration and remyelination [14–17].

CSPGs were recently identified by our group as a novel regulator of neuroinflammation in a mouse model of MS. In particular, the V1 isoform of versican (in which the core protein consists only of the GAGβ segment) is particularly abundant in inflammatory CNS lesions, and a mixture of lecticans stimulates bone marrow-derived macrophages to produce inflammatory cytokines and proteases [18]. Others have reported that the genetic loss of versican in lung macrophages attenuates their pro-inflammatory response to polyinosine-polycytidylic acid [19].

As CSPGs potently influence reparative processes in the CNS, it is important to explore approaches to overcome this impediment to regeneration. The biosynthetic process of CSPGs has largely been elucidated [20], and a number of pharmacological inhibitors of CSPG production have been examined for therapeutic effects, such as xylosides and fluorinated analogues that inhibit activity of CSPG biosynthesis enzymes [21, 22]. Following CNS insult, chondroitinase ABC (ChABC)-mediated CSPG digestion improved axonal sprouting, remyelination, and cognitive recovery [14–16, 23–27]. Perturbing CSPG signaling through antagonism of receptors (e.g., leukocyte-common antigen related and protein tyrosine phosphatase σ) also increased the population of alternatively activated macrophages following SCI [25]. However, finding an appropriate approach to removing deposited CSPGs in diffuse or heterogeneous pathology is difficult, and the regulators of CSPG production are understudied. Understanding the regulatory mechanisms of CSPG biosynthesis may provide more insight into limiting CSPG deposition after injury.

We interrogated the summary results from the 2011 MS genome-wide association screen (GWAS) and the 2013 MS Immunochip study (Immunochip 2013) focusing on regions containing genes involved in the metabolism of CSPGs, which led to the identification of the glycosyltransferase exostosin-like 2 (EXTL2) gene. EXTL2 is a glycosyltransferase that limits the production of CSPGs by terminating its synthesis [28]. Thus, we asked whether investigating the biology of EXTL2 and its effects on CSPG deposition and repair following a demyelinating injury to the spinal cord of mice could provide insights to endogenous targets for limiting CSPG production. Our results in mice with constitutive knockout of EXTL2 (EXTL2^−/−^) in the lysolecithin model of focal demyelination [29] highlight elevated CSPG deposition in lesions that contribute to the increased recruitment and activation of microglia/macrophages leading to the exacerbation of axonal loss.

## Methods

### Analyses of MS genetic databases

The MS GWAS performed jointly by the International Multiple Sclerosis Genetics Consortium (IMSGC) and the Wellcome Trust Case Control Consortium (WTCCC2) involved 9772 cases and 17,376 controls collected from across 15 different countries (all individuals were of European descent) [30]. The study generated data from 465,434 autosomal SNPs and identified 29 novel genome-wide significant associations. The MS Immunochip study [31] was a follow-up effort focused on genomic regions implicated in autoimmune disease GWAS. This study was performed by the IMSGC and involved 14,802 cases and 26,703 controls; there was no overlap of samples between these two studies. The Immunochip study was a focused follow-up effort which did not consider all parts of the genome, so some regions were more densely covered than others compared to the 2011 GWAS study. The Immunochip study generated data from 161,311 autosomal markers and identified 48 novel associations. Neither of the MS genetics studies considered the sex chromosomes and we were therefore unable to include sex-linked CSPG genes in this analysis.

Using our knowledge of CSPG biology, we identified a list of 154 autosomal genes (Table 1) involved intimately or remotely in the production and degradation of CSPGs. This includes proteoglycans (*Vcan*, *Acan*, *Ncan*, *Bcan*, *Bgn*, *Bsg*, *Gpc1-6*), degradation enzymes (*Adam*, *Adamts*, *Mmp*), and synthesis-related genes (*B3galt6*, *B3gat1-3*, *B4galt7*, *Chpf*, *Chpf2*, *Chst1-15*, *Chsy1*, *Chsy3*, *Csgalnact1-2*, *Ext1-2*, *Extl1-3*, *Fam20b*), among others.

**Table 1:** Names and description of genes associated with CSPG synthesis and turnover

Gene	Description
*Acan*	Aggrecan
*Aco1*	Aconitase 1, soluble
*Adam2*	ADAM metallopeptidase domain 2
*Adam7*	ADAM metallopeptidase domain 7
*Adam8*	ADAM metallopeptidase domain 8
*Adam9*	ADAM metallopeptidase domain 9
*Adam10*	ADAM metallopeptidase domain 10
*Adam11*	ADAM metallopeptidase domain 11
*Adam12*	ADAM metallopeptidase domain 12
*Adam15*	ADAM metallopeptidase domain 15
*Adam17*	ADAM metallopeptidase domain 17
*Adam18*	ADAM metallopeptidase domain 18
*Adam19*	ADAM metallopeptidase domain 19
*Adam20*	ADAM metallopeptidase domain 20
*Adam21*	ADAM metallopeptidase domain 21
*Adam22*	ADAM metallopeptidase domain 22
*Adam23*	ADAM metallopeptidase domain 23
*Adam28*	ADAM metallopeptidase domain 28
*Adam29*	ADAM metallopeptidase domain 29
*Adam30*	ADAM metallopeptidase domain 30
*Adam32*	ADAM metallopeptidase domain 32
*Adam33*	ADAM metallopeptidase domain 33
*Adamts1*	ADAM metallopeptidase with thrombospondin type 1 motif, 1
*Adamts2*	ADAM metallopeptidase with thrombospondin type 1 motif, 2
*Adamts3*	ADAM metallopeptidase with thrombospondin type 1 motif, 3
*Adamts4*	ADAM metallopeptidase with thrombospondin type 1 motif, 4
*Adamts5*	ADAM metallopeptidase with thrombospondin type 1 motif, 5
*Adamts6*	ADAM metallopeptidase with thrombospondin type 1 motif, 6
*Adamts7*	ADAM metallopeptidase with thrombospondin type 1 motif, 7
*Adamts8*	ADAM metallopeptidase with thrombospondin type 1 motif, 8
*Adamts9*	ADAM metallopeptidase with thrombospondin type 1 motif, 9
*Adamts10*	ADAM metallopeptidase with thrombospondin type 1 motif, 10
*Adamts12*	ADAM metallopeptidase with thrombospondin type 1 motif, 12
*Adamts14*	ADAM metallopeptidase with thrombospondin type 1 motif, 14
*Adamts15*	ADAM metallopeptidase with thrombospondin type 1 motif, 15
*Adamts16*	ADAM metallopeptidase with thrombospondin type 1 motif, 16
*Adamts17*	ADAM metallopeptidase with thrombospondin type 1 motif, 17
*Adamts18*	ADAM metallopeptidase with thrombospondin type 1 motif, 18
*Adamts19*	ADAM metallopeptidase with thrombospondin type 1 motif, 19
*Adamts20*	ADAM metallopeptidase with thrombospondin type 1 motif, 20
*App*	Amyloid beta (A4) precursor protein
*B3galt6*	UDP-Gal:betaGal beta 1,3-galactosyltransferase polypeptide 6
*B3gat1*	Beta-1,3-glucuronyltransferase 1 (glucuronosyltransferase P)
*B3gat2*	Beta-1,3-glucuronyltransferase 2 (glucuronosyltransferase S)
*B3gat3*	Beta-1,3-glucuronyltransferase 3 (glucuronosyltransferase I)
*B4galt7*	Xylosylprotein beta 1,4-galactosyltransferase, polypeptide 7
*Bcan*	Brevican
*Bgn*	Biglycan
*Bsg*	Basigin
*Chpf*	Chondroitin polymerizing factor
*Chpf2*	Chondroitin polymerizing factor 2
*Chst1*	Carbohydrate (keratan sulfate Gal-6) sulfotransferase 1
*Chst2*	Carbohydrate (N-acetylglucosamine-6-O) sulfotransferase 2
*Chst3*	Carbohydrate (chondroitin 6) sulfotransferase 3
*Chst4*	Carbohydrate (N-acetylglucosamine 6-O) sulfotransferase 4
*Chst5*	Carbohydrate (N-acetylglucosamine 6-O) sulfotransferase 5
*Chst6*	Carbohydrate (N-acetylglucosamine 6-O) sulfotransferase 6
*Chst7*	Carbohydrate (N-acetylglucosamine 6-O) sulfotransferase 7
*Chst8*	Carbohydrate (N-acetylgalactosamine 4-0) sulfotransferase 8
*Chst9*	Carbohydrate (N-acetylgalactosamine 4-0) sulfotransferase 9
*Chst10*	Carbohydrate sulfotransferase 10
*Chst11*	Carbohydrate (chondroitin 4) sulfotransferase 11
*Chst12*	Carbohydrate (chondroitin 4) sulfotransferase 12
*Chst13*	Carbohydrate (chondroitin 4) sulfotransferase 13
*Chst14*	Carbohydrate (N-acetylgalactosamine 4-0) sulfotransferase 14
*Chst15*	Carbohydrate (N-acetylgalactosamine 4-sulfate 6-O) sulfotransferase 15
*Chsy1*	Chondroitin sulfate synthase 1
*Chsy3*	Chondroitin sulfate synthase 3
*Cp*	Ceruloplasmin (ferroxidase)
*Csgalnact1*	Chondroitin sulfate N-acetylgalactosaminyltransferase 1
*Csgalnact2*	Chondroitin sulfate N-acetylgalactosaminyltransferase 2
*Dse*	Dermatan sulfate epimerase
*Ext1*	Exostosin glycosyltransferase 1
*Ext2*	Exostosin glycosyltransferase 2
*Extl1*	Exostosin-like glycosyltransferase 1
*Extl2*	Exostosin-like glycosyltransferase 2
*Extl3*	Exostosin-like glycosyltransferase 3
*Fam20b*	Family with sequence similarity 20, member B
*Fdx1*	Ferredoxin 1
*Fth1*	Ferritin, heavy polypeptide 1
*Ftl*	Ferritin, light polypeptide
*Ftmt*	Ferritin mitochondrial
*Galns*	Galactosamine (N-acetyl)-6-sulfate sulfatase
*Gpc1*	Glypican 1
*Gpc2*	Glypican 2
*Gpc3*	Glypican 3
*Gpc4*	Glypican 4
*Gpc5*	Glypican 5
*Gpc6*	Glypican 6
*Gsta1*	Glutathione S-transferase alpha 1
*Gsta2*	Glutathione S-transferase alpha 2
*Gsta3*	Glutathione S-transferase alpha 3
*Gsta4*	Glutathione S-transferase alpha 4
*Gsta5*	Glutathione S-transferase alpha 5
*Gstk1*	Glutathione S-transferase kappa 1
*Gstm1*	Glutathione S-transferase mu 1
*Gstm2*	Glutathione S-transferase mu 2 (muscle)
*Gstm3*	Glutathione S-transferase mu 3 (brain)
*Gstm4*	Glutathione S-transferase mu 4
*Gstm5*	Glutathione S-transferase mu 5
*Gstp1*	Glutathione S-transferase pi 1
*Gstt1*	Glutathione S-transferase theta 1
*Gstt2*	Glutathione S-transferase theta 2
*Hamp*	Hepcidin antimicrobial peptide
*Hapln1*	Hyaluronan and proteoglycan link protein 1
*Hapln2*	Hyaluronan and proteoglycan link protein 2
*Has1*	Hyaluronan synthase 1
*Has2*	Hyaluronan synthase 2
*Has3*	Hyaluronan synthase 3
*Heph*	Hephaestin
*Hmox1*	Heme oxygenase (decycling) 1
*Hmox2*	Heme oxygenase (decycling) 2
*Hp*	Haptoglobin
*Hpr*	Haptoglobin-related protein
*Ilf3*	Interleukin enhancer binding factor 3, 90 kDa
*Ireb2*	Iron-responsive element binding protein 2
*Ltf*	Lactotransferrin
*Mfi2*	Antigen p97 (melanoma associated) identified by monoclonal antibodies 133.2 and 96.5
*Mgst1*	Microsomal glutathione S-transferase 1
*Mgst2*	Microsomal glutathione S-transferase 2
*Mgst3*	Microsomal glutathione S-transferase 3
*Mmp1*	Matrix metallopeptidase 1 (interstitial collagenase)
*Mmp2*	Matrix metallopeptidase 2 (gelatinase A, 72 kDa gelatinase, 72 kDa type IV collagenase)
*Mmp3*	Matrix metallopeptidase 3 (stromelysin 1, progelatinase)
*Mmp7*	Matrix metallopeptidase 7 (matrilysin, uterine)
*Mmp8*	Matrix metallopeptidase 8 (neutrophil collagenase)
*Mmp9*	Matrix metallopeptidase 9 (gelatinase B, 92 kDa gelatinase, 92 kDa type IV collagenase)
*Mmp10*	Matrix metallopeptidase 10 (stromelysin 2)
*Mmp11*	Matrix metallopeptidase 11 (stromelysin 3)
*Mmp12*	Matrix metallopeptidase 12 (macrophage elastase)
*Mmp13*	Matrix metallopeptidase 13 (collagenase 3)
*Mmp14*	Matrix metallopeptidase 14 (membrane-inserted)
*Mmp15*	Matrix metallopeptidase 15 (membrane-inserted)
*Mmp16*	Matrix metallopeptidase 16 (membrane-inserted)
*Mmp17*	Matrix metallopeptidase 17 (membrane-inserted)
*Mmp19*	Matrix metallopeptidase 19
*Mmp20*	Matrix metallopeptidase 20
*Mmp21*	Matrix metallopeptidase 21
*Mmp23b*	Matrix metallopeptidase 23B
*Mmp24*	Matrix metallopeptidase 24 (membrane-inserted)
*Mmp25*	Matrix metallopeptidase 25
*Mmp26*	Matrix metallopeptidase 26
*Mmp27*	Matrix metallopeptidase 27
*Mmp28*	Matrix metallopeptidase 28
*Ncan*	Neurocan
*Ndst1*	N-deacetylase/N-sulfotransferase (heparan glucosaminyl) 1
*Ndst2*	N-deacetylase/N-sulfotransferase (heparan glucosaminyl) 2
*Slc11a1*	Solute carrier family 11 (proton-coupled divalent metal ion transporter), member 1
*Slc11a2*	Solute carrier family 11 (proton-coupled divalent metal ion transporter), member 2
*Slc39a14*	Solute carrier family 39 (zinc transporter), member 14
*Slc40a1*	Solute carrier family 40 (iron-regulated transporter), member 1
*Tf*	Transferrin
*Tfr2*	Transferrin receptor 2
*Tfrc*	Transferrin receptor
*Timp3*	TIMP metallopeptidase inhibitor 3
*Ust*	Uronyl-2-sulfotransferase
*Vcan*	Versican
*Xylt1*	Xylotransferase I
*Xylt2*	Xylotransferase II

### Animals

All experiments were performed in accordance with the ethical guidelines of the Animal Care Committee at the University of Calgary. Female wild-type C57Bl/6 mice aged 6 to 8 weeks were purchased from Charles River (Montreal) and Extl2^−/−^ mice were previously described [28, 32] and bred at the University of Calgary Animal Health Unit. Experiments conducted with C57Bl/6 wild-type mice from our in-house bred colony or purchased from Charles River did not differ in responses when directly compared. The Extl2-null mice were generated by truncating exon 3 and removal of exon 4 of the *Extl2* gene [32]. No defects in breeding or physical impairments were published for the *Extl2*^−/−^ mice, and we did not observe any obvious abnormalities including weight changes in our colony transferred from Japan through co-author Hiroshi Kitagawa. All experiments were completed on female mice aged 8 to 12 weeks.

### Lysolecithin-induced demyelination

Experimental demyelination was produced in the ventral spinal cord by stereotactic injection of the toxin lysolecithin [29]. A 10 μL glass Hamilton syringe with a 34-gauge needle was preloaded with a 1% solution of lysolecithin. Animals were anesthetized with a mixture of ketamine/xylazine (100 mg/kg and 10 mg/kg, respectively) administered through an intraperitoneal injection. The analgesic buprenorphine (0.05 mg/kg) was injected subcutaneously prior to surgery. Ophthalmic lubricant was applied to the eyes to prevent them from drying during surgery. A small area on the back was shaved with a razor and the surgical field was disinfected with 70% ethanol and iodine solution. A midline incision was made, and underlying muscle and adipose tissue separated using retractors. Connective tissue between the T3 and T4 vertebra was cut to reveal the spinal cord. The dura mater was opened using a 30-gauge needle. The tip of the Hamilton 34-gauge needle was placed at the surface of the spinal cord, lateral to midline. A baseline measurement was made, and 1.3 mm was subtracted from the baseline measurement to obtain the final desired measurement for targeting the ventral white matter. The needle was depressed and injection of lysolecithin was completed with one rotation of the micromanipulator every 5 s for 2 min, for a final volume of 0.5 μL. Animals were sutured and placed in a recovery chamber and monitored until awake. Postoperative buprenorphine was administered subcutaneously at 0.05 mg/kg 16 h following surgery.

### Tissue harvesting and processing

Animals were sacrificed at 7, 14, and 21 days post-injection of lysolecithin. Mice were given a lethal dose of ketamine/xylazine anesthetic. Animals were perfused through the left ventricle of the heart with 15 mL of phosphate-buffered saline (PBS) and 15 mL of 4% paraformaldehyde (PFA). The cervical and upper thoracic section of the spinal cord was harvested. Spinal cords were laid on strips of thick card stock to maintain structural integrity. For western blot, spinal cords were immediately flash frozen in liquid nitrogen and stored at – 80 °C until use. For immunohistochemistry, spinal cords were post-fixed overnight in 4% PFA at 4 °C, then transferred into 30% sucrose solution for 72 h. Spinal cords were then frozen into O.C.T. blocks using dry ice and blocks were stored at – 20 °C until sectioning on the cryostat.

### Immunohistochemistry

Spinal cords frozen in OCT blocks were cut into 20 μm-thick sections and mounted on glass slides. Staining of tissue sections was done in a humidity chamber to prevent evaporation. One section per spinal cord was chosen for staining and analysis. Sections were selected based on proximity to the lesion epicenter; the latter was elucidated as the section in a given mouse containing the largest lesion as determined using eriochrome cyanine staining. The adjacent sections were taken for immunofluorescent staining. This selection of tissue sections is similar to our analyses of other features described in previous publications (e.g., [21]).

All washes were completed using PBS containing 0.25% Tween-20. Blocking of tissue sections was done using horse serum blocking solution (0.01 M PBS, 10% horse serum, 1% bovine serum albumin (BSA), 0.1% cold fish skin gelatin, 0.1% Triton-X100, and 0.05% Tween-20) for 2 h at room temperature. Blocking solution was removed prior to application of primary antibodies. Primary antibodies were diluted in 0.01 M PBS containing 1% BSA, 0.1% cold fish skin gelatin, and 0.5% Triton-X100. Primary antibodies were obtained from various sources and used at dilutions detailed as follows: versican V0/V1 (Millipore, ab1033, 1:200), myelin basic protein (MBP, Abcam, ab7349, 1:1000), Olig2 (Millipore, AB9610, 1:200), platelet-derived growth factor receptor α (PDGFRα, R&D Systems, AF1062, 1:100), adenomatous polyposis coli (APC, Millipore, clone CC-1, OP80, 1:100), neurofilament heavy chain 200 kD (NF-200, EnCor, RPCA-NF-H, 1:1000), ionized calcium-binding adaptor molecule 1 (Iba1, Wako, 019-19741, 1:1000), CD16/32 (Bio-Rad, MCA1075, 1:200), and CD206 (R&D Systems, AF2535, 1:200). Primary antibodies were left on sections overnight at room temperature. Secondary antibodies were diluted in the same dilution buffer as primary antibodies and left for 2 h at room temperature. Slides were mounted with Fluoromount G® (SouthernBiotech). Fluorescence imaging was done using the Leica TCS SP8 confocal laser scanning microscope.

Versican exists as 4 isoforms: V0, V1, V2, and V3. The antibody to versican V1 (rabbit anti-mouse versican V0/V1, Millipore, AB1033) recognizes the GAG beta domain, and the versican V2 antibody (rabbit anti-mouse versican V0/V2, Millipore, AB1032) recognizes the GAG alpha domain, both of which are present in the versican V0 splice isoform. However, due to the lack of colocalization of versican V1/V0 and versican V2/V0 in similar regions (data not shown), we attributed the staining of versican V1 to primarily V1 isoform, although this is displayed in figures as V0/V1.

### Chondroitinase ABC digestion

Chondroitinase ABC (ChABC) digestion was done for immunohistochemical staining of V0/V1, as the removal of the GAG chains facilitates the binding of antibody to the core protein. Slides were thawed to room temperature and washed once with PBS. ChABC was diluted in PBS and applied to slides at a working concentration of 0.2 units/mL. Slides were incubated in a heated chamber at 37 °C for 30 min. Slides were washed with PBS before proceeding with staining.

### Bone marrow-derived macrophages (BMDM) isolation and culture

C57Bl/6 wild-type or EXTL2^−/−^ animals were euthanized by overdose of ketamine/xylazine. Femurs and tibia were dissected from the body and soaked briefly in ethanol. Bone marrow was harvested from bones and centrifuged at 1200 rpm for 10 min. Cells were resuspended in PBS and counted. Cells were centrifuged again at 1200 rpm for 10 min. BMDMs were cultured at 37 °C, 8.5% CO_2_ in 100 mm plastic, uncoated, tissue culture-treated dishes in Dulbecco’s modified eagle’s medium (DMEM) containing 10% FBS, 1% penicillin-streptomycin (Gibco), 1% sodium pyruvate (Gibco), 1% GlutaMAX™ (Gibco), and 10% L929 conditioned media. Cells were cultured for 8–10 days before harvesting. A half-volume media change was done on day 5 and full media change was done on day 7. To harvest BMDMs, media was removed from plates and cold PBS was applied to the dishes. Cell scrapers were used to dissociate cells from dishes. Cells were collected in 50 mL tubes and centrifuged at 1200 rpm for 10 min, then resuspended in same media, except lacking L929 conditioned media. Cells were replated at a density of 50,000 cells/100 μL/well in a 96-well plate. Plates were incubated at 37 °C in 5% CO_2_. A media switch to serum-free DMEM with supplements was performed at 24 h post-plating, and treatments were added to wells in 1% FBS in DMEM.

### Enzyme-linked immunosorbent assay

Supernatant from BMDM cultures (as described above) was harvested at 24 h following the initiation of stimulation. Supernatant was frozen at − 80 °C until use. Samples were thawed to room temperature and ELISA was performed according to manufacturer instructions (Invitrogen).

### Mouse MMP 5-plex Luminex assay

BMDM supernatant was harvested at 24 h following the initiation of stimulation. Supernatant was frozen at − 80 °C until the assay was performed. Tubes were stored on ice until used. Samples were processed by Eve Technologies (Calgary) for a panel of 5 MMP members.

### Oligodendrocyte precursor cell isolation and harvest

P0-2 CD-1 mouse pups were sacrificed, and brains were isolated. Under a dissection microscope, meninges and choroid plexus tissue were removed from the cortical and ventricular surfaces, respectively. Cortices were dissected away from the hippocampus and cerebellum and cut into 2–3 mm^2^ pieces. A cocktail of papain, DNase, and L-cysteine was used for tissue digestion. The digestion cocktail was added to tissue in a 50-mL tube and the tube was placed in a 37 °C water bath for 30 min, with occasional inversion. More DNase was added if tissue became gel-like. Cells were centrifuged at 1200 rpm for 10 min and resuspended in growth medium. Dissociated cells were plated in T-75 culture flasks pre-coated with poly-L-lysine (10 μg/mL, Sigma). The mixed glial culture was maintained in DMEM containing 10% FBS, 1% GlutaMAX™, 1% sodium pyruvate, and 1% penicillin-streptomycin. Flasks were kept at 37 °C, 8.5% CO_2_ for 9 days. Half media changes were performed every 3 days. On day 9, flasks were shaken overnight in a 37 °C, 5% CO_2_ incubator at 220 rpm to dislodge OPCs and microglia from astrocytes. The supernatant containing OPCs and microglia was plated on plastic, uncoated 100 mm dishes for 30 min to allow microglia to adhere. Media containing enriched OPCs was harvested and plated in oligodendrocyte differentiating media (described below) at 5000–10,000 cells/100 μL/well in a 96-well plate pre-coated with poly-L-lysine and other extracellular matrix molecules when appropriate.

Oligodendrocyte differentiating medium was DMEM containing 2% (v/v) B27 supplement (Gibco), 1% (v/v) oligodendrocyte supplement cocktail (see below), 1% (v/v) GlutaMAX™ (Gibco), 100 μm sodium pyruvate (Gibco), 1% (v/v) penicillin-streptomycin (Gibco), 50 μg/mL holo-transferrin (Sigma), 5 μg/mL N-acetyl-L-cysteine (Sigma), 50 ng/mL ciliary neurotrophic factor (PeProTech), and 0.005% (v/v) Trace Elements B (Corning).

Oligodendrocyte supplement cocktail was made from 100 mL DMEM with 1% BSA, 0.06% progesterone (Sigma), 16.1% putrescine (Sigma), 0.005% sodium selenite (Sigma), 0.4% 3,3′,5-triiodo-L-thyronine (Sigma).

### Neuron culture and harvest

E16-18 C57Bl/6 pups were obtained from pregnant C57Bl/6 mice purchased from Charles River. The pregnant mouse was anesthetized with a mixture of ketamine and xylazine and the uterine horns were dissected. Pups were sacrificed by decapitation and brains were isolated. Meninges were microdissected from the brain and cortices were cut into small pieces and collected. For tissue digestion, harvested brain tissue was suspended in HBSS (Gibco) containing 0.025% trypsin. The suspension was immersed in a 37 °C water bath with a stir bar to homogenize tissue. Following digestion, heat inactivated FBS was added to inactivate trypsin and cells were filtered into a 50 mL tube and centrifuged at 1200 rpm for 10 min. The supernatant was discarded, and cells were then washed one time with HBSS and centrifuged as before. The supernatant was discarded, and cells were resuspended in growth medium comprised of Neurobasal Plus Medium (Gibco) containing 2% B27 Plus supplement (Gibco). Cells were seeded at 1 to 2 × 10^7^ cells per T-25 flasks coated with poly-L-ornithine. Cells were grown in a 37 °C incubator in 5% CO_2_ for at least 24 h in the T-25 flask before they were harvested using 0.025% trypsin. Cells were then plated at a density of 100,000 cells/well in a 96-well plate coated with poly-L-ornithine in the previously described growth medium. Neurons were allowed to grow for 24 h prior to treatment with BMDM conditioned media.

### Cell staining and imaging

All washing was done using PBS. Plates were placed on an orbital shaker during all incubations. For OPCs, blocking was done using 10% normal goat serum (NGS) in PBS or Odyssey® Blocking Buffer (Licor) for 1 h at room temperature. Primary antibody against O4 (Millipore) was diluted 1:50 in blocking buffer and incubated for 2 h at room temperature or overnight at 4 °C. An anti-mouse IgM antibody conjugated to Alexa Fluor® 488 (Jackson ImmunoResearch) as well as nuclear yellow was added in blocking buffer for 1 h at room temperature. Plates were washed with PBS and imaged using the ImageXpress Micro XLS Widefield High-Content Analysis System (Molecular Devices).

For BMDMs, for detection of intracellular molecules, cells were permeabilized using 0.025% Triton-X100 for 5 min at room temperature. No permeabilization was required for surface molecules. Blocking was done using Odyssey® Blocking Buffer (Licor) for 30 min at room temperature. Primary antibodies were diluted in the same blocking buffer incubated overnight at 4 °C. Secondary antibodies were diluted in the same blocking buffer and incubated for 1 h at room temperature. Nuclear yellow was added in PBS and incubated for 10 min. Plates were washed with PBS and imaged using the ImageXpress Micro XLS Widefield High-Content Analysis System.

### Image analysis

For immunohistochemistry, images were processed and analyzed using ImageJ. All images were blinded prior to analysis. For percent of lesion area analyses, lesion area was demarcated using MBP staining. A disruption in MBP density was determined to be lesional. The lesion area was isolated and area of signal of interest was determined using differential thresholding. The area occupied by the signal was normalized to total area of the lesion or represented as an absolute area. For axonal counts in the lesion, the lesion area was demarcated using MBP. Differential thresholding was used to isolate individual axons present inside the lesional area and was automatically counted. The number of axons within the lesion was normalized to total lesion area to obtain density. For oligodendrocyte-lineage cell counting and scoring, the same approach to demarcating the lesion area was used. Images containing Olig2^+^ nuclei were overlaid with either PDGFRα staining or CC1 staining. Colocalization of either PDGFRα or CC1 with Olig2 was determined to be an OPC or mature oligodendrocyte, respectively. The density of oligodendrocytes in the lesions was calculated by isolating the lesion area and counting the number of Olig2^+^ nuclei and normalizing counts to the respective lesion area. Percentage of OPCs or mature oligodendrocytes was calculated using the total number of Olig2^+^ nuclei. We assessed the mean fluorescence intensity of the macrophage activation markers CD16/32 and CD206 within the lesion, internally normalized to the background fluorescence. Mean fluorescence intensity was assessed within the area of positive immunoreactivity.

For immunocytochemistry, images were analyzed on the MetaXpress High-Content Image Acquisition and Analysis Software (Molecular Devices, San Jose, CA). Appropriate thresholds were determined for true signals. OPC cultures were analyzed using the Neurite Outgrowth program, and BMDM cultures were analyzed using the Cell Scoring program. Both processing protocols are from Molecular Devices.

### Statistical analysis

All statistical analysis was performed with GraphPad Prism 8.0. Student’s *t* test, ordinary one-way ANOVA, and two-way ANOVA statistical tests were used with an alpha of 0.05. Individual tests and post hoc tests are described in figure legends. For assessment of lesions with respect to versican V1 deposition, oligodendrocytes, lesion area, macrophage/microglia, and axons, between 4 and 12 individual animals were used, and one section per lesion was quantified. The individual performing the surgery was not blinded to the genotype of animals; however, images were blinded prior to analysis on the ImageJ software. Each animal/lesion was considered an *n* of 1. In some cases, the quality of the tissue section or the staining was not deemed adequate for blinded analysis, and these were omitted. For lesion area analysis using MBP staining, animals from two sets of surgeries were combined. Other immunohistological analyses were performed on one of the two sets of surgeries.

BMDMs were derived from 1 to 2 wild-type or EXTL2^−/−^ mice. OPCs were derived from between 8 to 12 CD-1 pups. Neurons were derived from 8 to 12 C57Bl/6 prenatal pups aged E13-15. For all cell culture experiments, each data point was derived from one well. Subsequent replicates of experiments were performed and reported in figure captions.

## Results

### SNP data mining from MS databases implicates EXTL2

Ignoring pseudogenes, we identified 154 autosomal genes involved in the metabolism of CSPGs (Table 1). Several of these genes map close to each other so after allowing a 100-kb extension from both ends, the 154 genes were found to map to 125 distinct genomic regions containing 7382 SNPs in the GWAS (median 42 SNPs per region, range 7–291) and 3139 SNPs (from 118 of the 125 regions) in the Immunochip (median 5 SNPs per region, range 1–494).

Both the GWAS and the Immunochip studies included secondary analyses of disease severity, as measured by the Multiple Sclerosis Severity Score and the clinical course (relapse onset vs progressive onset). None of these secondary analyses identified significant associations so it was unsurprising that no enrichment for association was seen among the SNPs mapping close to CSPG-related genes.

Six of the SNPs from the GWAS showed genome-wide significant evidence for association with all of these SNPs mapping to the region containing EXTL2. These SNPs all showed concordant effects on risk in the Immunochip study with significant but less extreme *p* values (ranging from 1.4e− 4 to 3.7e− 6). All six of the SNPs mapping to the region containing the EXTL2 gene were confirmed as associated with genome-wide significance in the larger MS GWAS meta-analysis (IMSGC 2017 BioRxiv 143933) that involved a total of 14,802 cases and 26,703 controls, including those considered in the 2011 GWAS. Combining all available genetic data showed that there are likely at least two independent MS susceptibility variants in the region of the EXTL2 gene (IMSGC 2017 BioRxiv 143933); 5 (rs12746227, rs11809572, rs11581062, rs11588568, and rs7541397) of the 6 SNPs were identified tag one of these effects and the remaining SNP (rs7552544) tags the second. In the final combined analysis, there was highly significant evidence for association with MS in this region (*p* = 1.1e− 22). These data thus confirm beyond any reasonable doubt that genetic variation in this region influences susceptibility to MS; however, it is important to note that EXTL2 is not the only gene in the region of these associated SNPs. It is therefore not possible to conclude with certainty that these variants exert their effects on risk via EXTL2 rather some other gene within the region. Nonetheless, the genetic analyses provided a solid rationale to evaluate the biology of EXTL2 in models of MS.

### Increased deposition and persistence of versican V1 and CS-A glycosaminoglycan following injury in the EXTL2^−/−^ spinal cord

Based on previous literature suggesting that EXTL2 is a negative regulator of CSPG synthesis [32], we first asked whether loss of EXTL2 results in increased CSPG deposition into an injured area during the reparative and remyelinating phases. We induced lysolecithin lesions in the ventral columns of spinal cords of wild-type and EXTL2^−/−^ animals and evaluated total CS-A (not shown) and versican V1 deposition into the lesion at 14 and 21 days post-lesion (dpl). Versican V1 was focused upon since this was the predominant lectican CSPG member produced after lysolecithin injury [21].

**Fig. 1:**
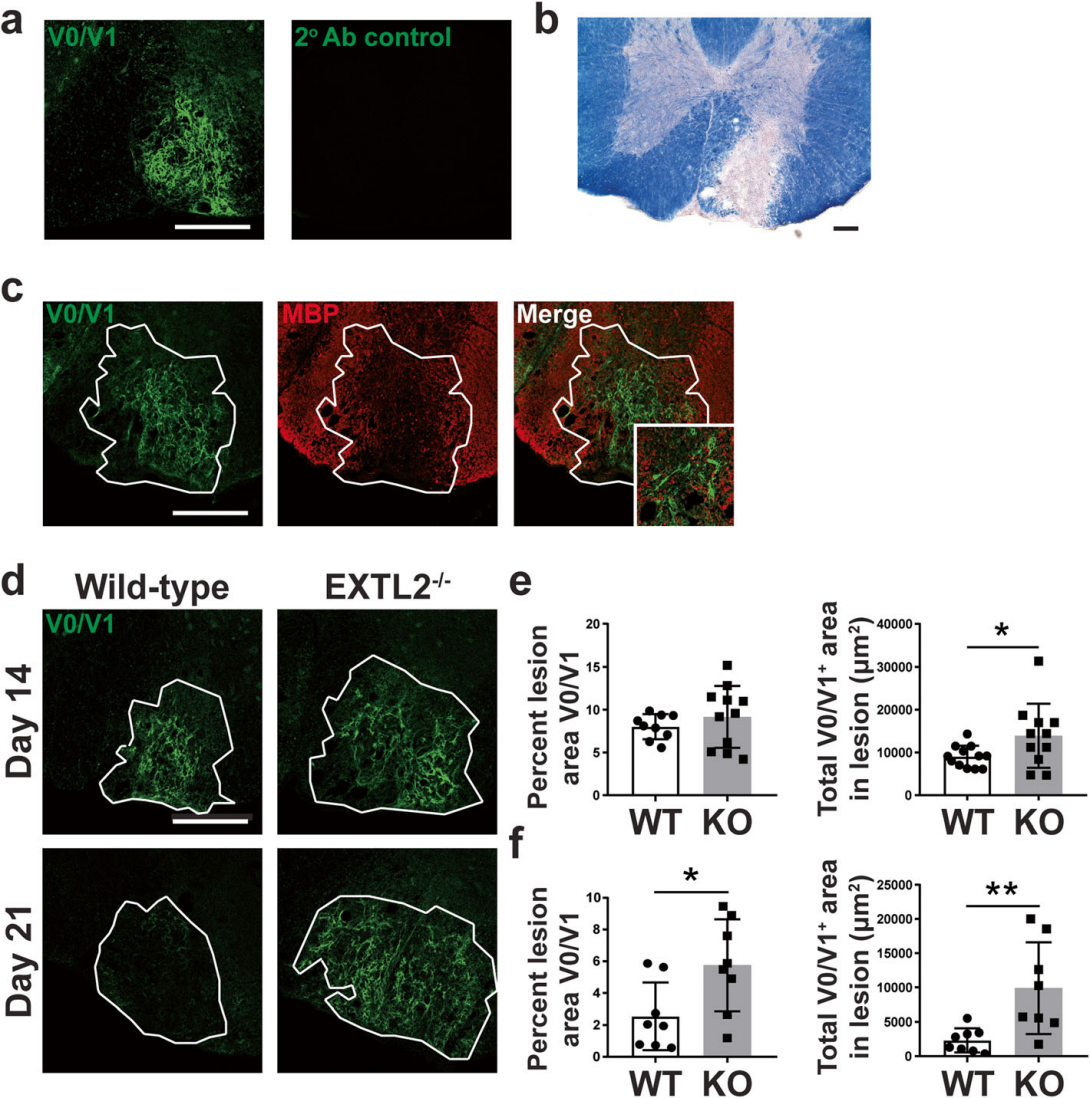
Immunolabeling of versican V0/V1 in wild-type and EXTL2^−/−^ lesions. **a** Representative images of tissue sections stained with the anti-versican V0/V1 antibody (left) and the secondary antibody control. Scale bar = 200 μm. **b** Representative lesion from wild-type day 14 animal stained with eriochrome cyanine R (scale bar = 100 μm) and **c** immunofluorescence staining for versican V0/V1, MBP, and merged image. Scale bar on immunofluorescent image = 200 μm. Inset shows high magnification image of versican and MBP. **d** Representative images of versican V0/V1 in wild-type and EXTL2^−/−^ lesions at 14 and 21 dpl. White outline indicates approximate lesion area. Scale bar = 200 μm. Quantification of versican area within the lesions, in terms of percent area of the whole lesion determined separately by MBP staining, and in terms of absolute area within the isolated lesion are shown in **e** for day 14 and **f** for day 21. Graphs show mean with standard deviation. Two-tailed, unpaired Student’s *t* test. *p < 0.05, **p < 0.01

Eriochrome cyanine staining shows a demarcated area in the ventral column of the spinal cord with distinctive loss of myelin in the lesion, corresponding to an increase in V1 immunoreactivity (Fig. 1a, b). In both wild-type and EXTL2^−/−^ spinal cords, upregulation of V1 was limited to the lesion site (Fig. 1c). By 14 dpl, there was a greater accumulation of V1 in EXTL2^−/−^ lesions compared to wild-type (Fig. 1d, e). At day 21, V1 was largely cleared from the wild-type lesion, whereas it remained in the lesion of EXTL2^−/−^ animals (Fig. 1d, f).

### EXTL2^−/−^ mice exhibit no overt differences in OPC repopulation and maturation following injury

**Fig. 2:**
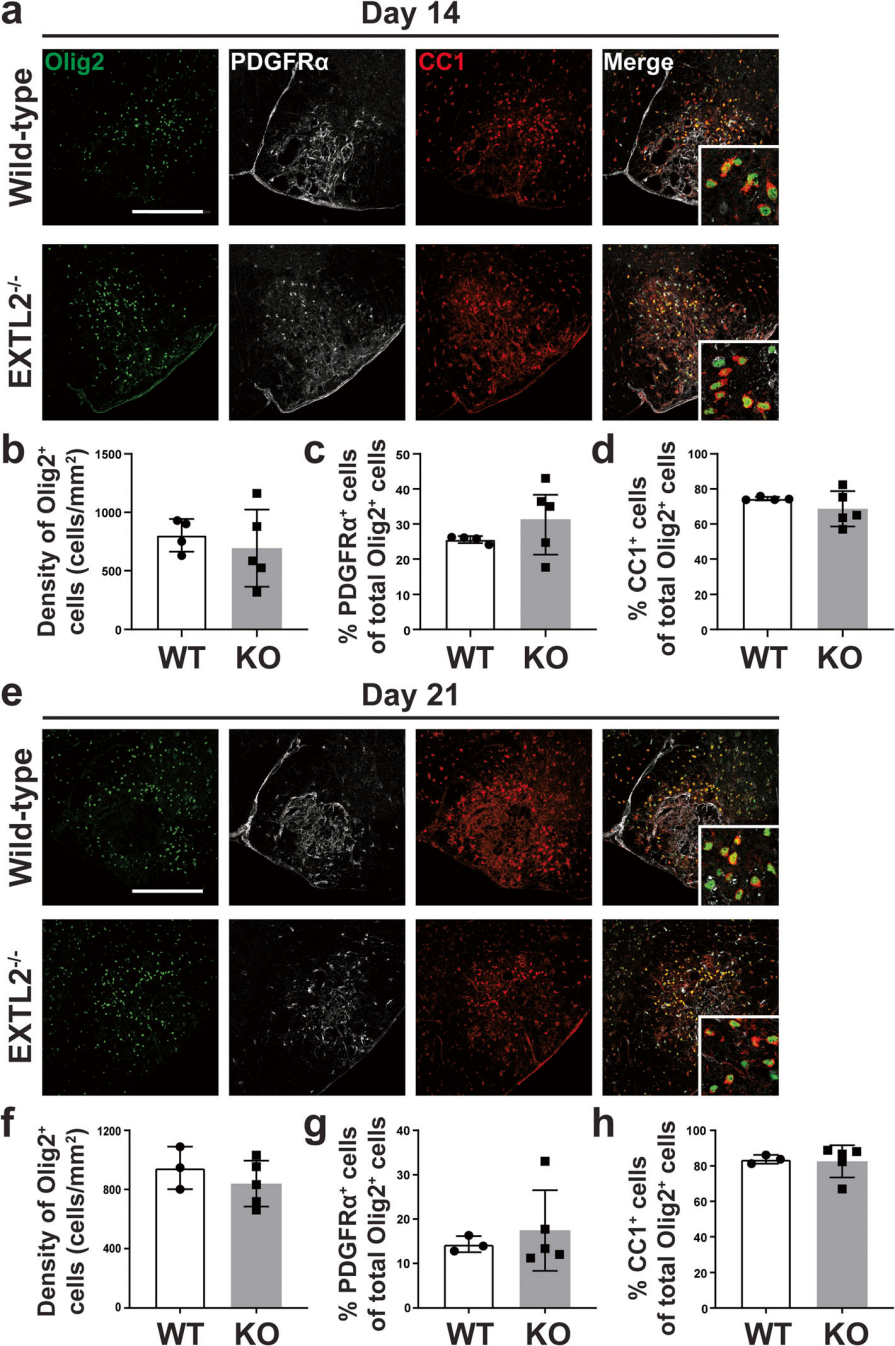
Immunostaining and quantification of oligodendrocyte lineage cells following injury. Representative images of lesions labeled with Olig2, PDGFRα, and APC (CC1) are shown in **a** and **e**, for days 14 and 21, respectively. Scale bar = 200 μm. Insets are high magnification images of individual Olig2^+^ nuclei co-labeled with PDGFRα or APC. Density of all Olig2^+^ nuclei are quantified for day 14 in **b** and day 21 in **f**. Proportion of Olig2^+^PDGFRα^+^ OPCs at both day 14 and 21, calculated as a fraction of all Olig2^+^ cells, are shown in **c** and **g**; **c** shows day 14 and **g** shows day 21 analyses. Graphs show mean with standard deviation. Two-tailed, unpaired Student’s *t* test

We next asked whether the extensive deposition and persistence of CSPGs in the EXTL2^−/−^ animals impacted the remyelination process. As there is extensive literature on the inhibitory nature of CSPGs on OPCs, we expected a deficiency or delay in remyelination in the EXTL2^−/−^ mice. In the spinal cord lysolecithin model, OPCs loss occurs in the acute phase of injury and they then repopulate the lesion after 3–4 days, with subsequent accumulation of OPCs and eventual differentiation into mature oligodendrocytes and remyelination ([29]; Jensen et al., 2018). We analyzed spinal cord sections stained for the transcription factor Olig2 that defines cells of the oligodendrocyte lineage, the surface receptor PDGFRα that is selective to OPCs, and APC (adenomatous polyposis coli, also called CC1, labeling mature oligodendrocytes) to determine the number of oligodendrocyte-lineage cells present within the lesion at days 14 and 21 post-injury (Fig. 2a, e). Unexpectedly, we found no significant differences in the density of Olig2^+^ oligodendrocyte-lineage cells within the lesion (Fig. 2b, f), or in proportions of Olig2^+^PDGFRα^+^ OPCs (Fig. 2c, g) and Olig2^+^APC^+^ oligodendrocytes (Fig. 2d, h) at any of the assessed time points. No deficiencies in OPC recruitment to the lesion, nor maturation into oligodendrocytes, was apparent by day 14 and day 21 after lysolecithin injury.

### Exacerbated lesion area and decreased axonal density in EXTL2^−/−^ lesions

**Fig. 3:**
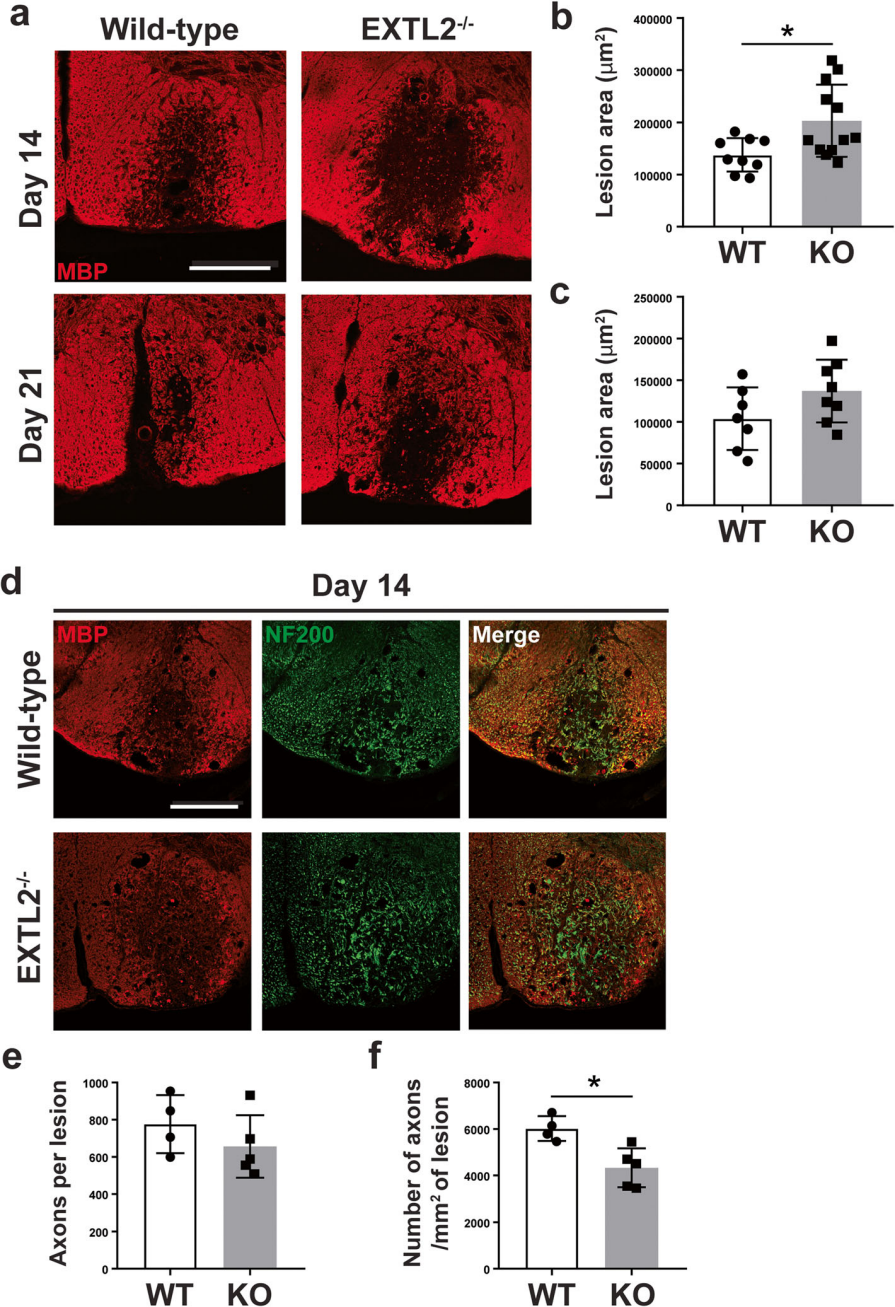
Assessment of area of lesion by loss of MBP immunoreactivity and changes in axonal density in wild-type vs EXTL2^−/−^ lesions at day 14. **a** Representative images of MBP immunofluorescent staining in wild-type and EXTL2^−/−^ mice. Scale bar = 200 μm. Lesion area determined by loss of MBP immunoreactivity was quantified for both day 14 (**b**) and day 21 (**c**). Two-tailed, unpaired Student’s *t* test. **d** Representative images from day 14 lesions labeled with MBP and NF200 to denote demyelinated area and axons, respectively. Scale bar = 200 μm. **e** Total number of axons per lesion. **f** Density of axons calculated by dividing number of axons over lesion area. Graphs show mean with standard deviation. Two-tailed, unpaired Student’s *t* test. *p < 0.05

In our characterization of the EXTL2^−/−^ phenotype following injury, we found an exacerbated injury area demarcated by loss of MBP immunoreactivity (Fig. 3a). Analysis of the total area of MBP disruption in both wild-type and EXTL2^−/−^ lesioned spinal cords showed a larger area of demyelination in EXTL2^−/−^ spinal cords at day 14 (Fig. 3b) and a similar but non-statistically significant trend at day 21 (Fig. 3c) following injection of lysolecithin compared to those of wild-type animals. We then hypothesized that there could be a corresponding loss of axons in the demyelinated area. To identify axons, we used an antibody against the 200 kD neurofilament (NF200), which reliably labels axons in the ventral column (Fig. 3d). Qualitatively, there was close correlation between the area of axonal loss to area of demyelination (Fig. 3d). At day 14, we observed a decreased axonal density in the EXTL2^−/−^ lesions, corroborating our finding of a larger area of demyelination (Fig. 3e, f). Lesions from day 21 animals had too much background to be evaluated for axons. Overall, we observed a larger lesion and a greater loss of axons in the EXTL2^−/−^ spinal cord with injection of lysolecithin.

### Increased accumulation of microglia and macrophages within lesions of EXTL2^−/−^ animals

**Fig. 4:**
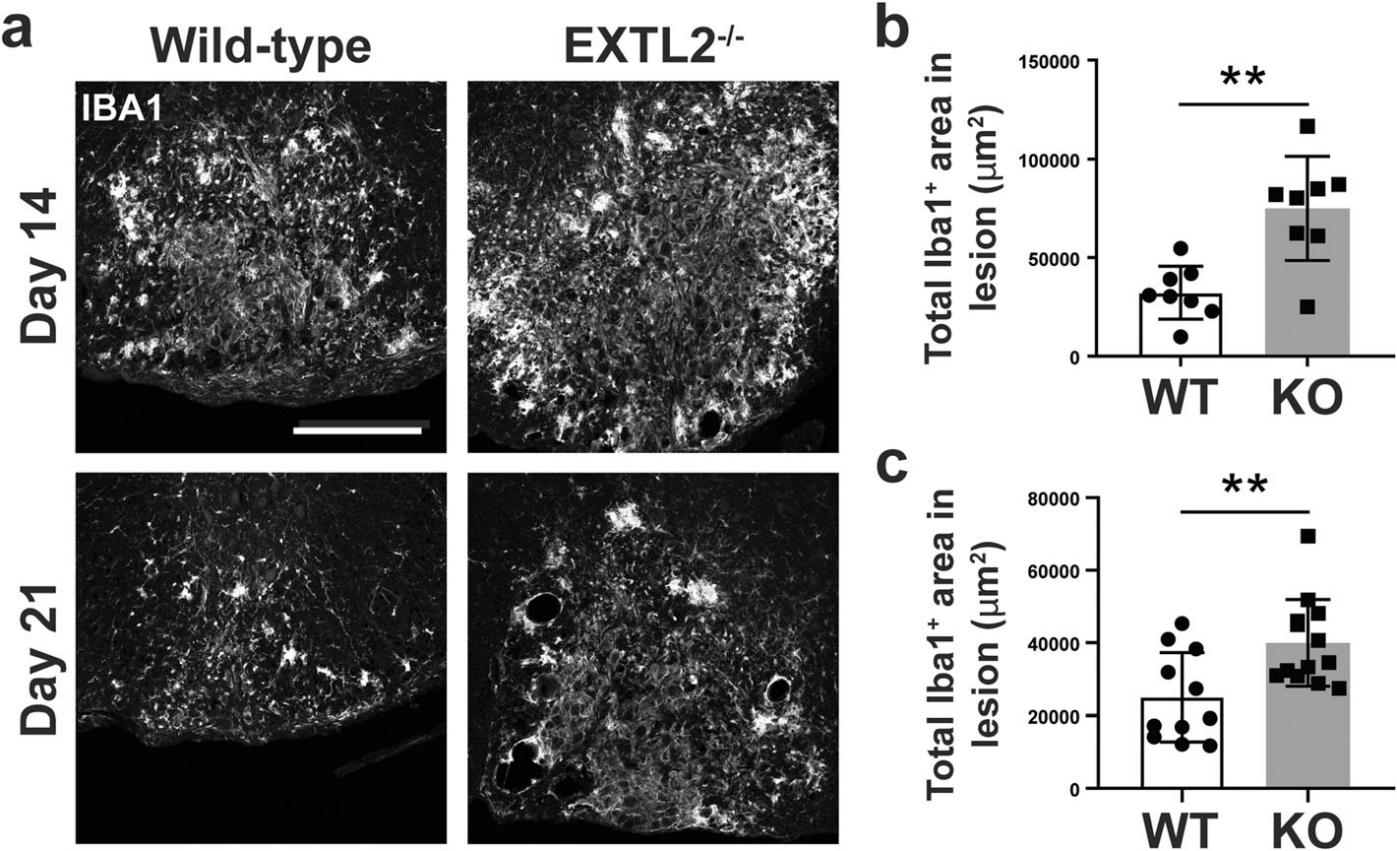
Staining and quantification of Iba1 immunoreactivity in the lesion. Iba1 staining is shown in representative images for day 14 and day 21 in **a**. Scale bar = 200 μm. Comparison of total Iba1^+^ area within the lesion in wild-type and EXTL2^−/−^ mice for day 14 (**b**) and day 21 (**c**) are shown. Graphs show mean with standard deviation. Two-tailed, unpaired Student’s *t* test. **p < 0.01

Given our previous report of CSPGs being a stimulator of macrophages in EAE [18] and our previous finding of exacerbated injury in the EXTL2^−/−^ spinal cord, we investigated whether there were corresponding pro-inflammatory phenotypes in lesional microglia/macrophages in EXTL2^−/−^ animals. Macrophages and microglia were labeled by Iba1 (ionized calcium-binding adaptor molecule 1) (Fig. 4a). We determined that there was a greater area of lesion covered by Iba1 immunoreactivity at day 14 (Fig. 4b), indicating an increase in macrophages/microglia density within the lesion, in the EXTL2^−/−^ animals. Greater Iba1 immunoreactivity was still observed at day 21 (Fig. 4c), suggesting macrophages/microglia persisted for longer in the EXTL2^−/−^ lesions.

### No difference in the surface marker expression of CD16/32 and CD206 in lesional Iba1^+^ cells in wild-type and EXTL2^−/−^ mice

**Fig. 5:**
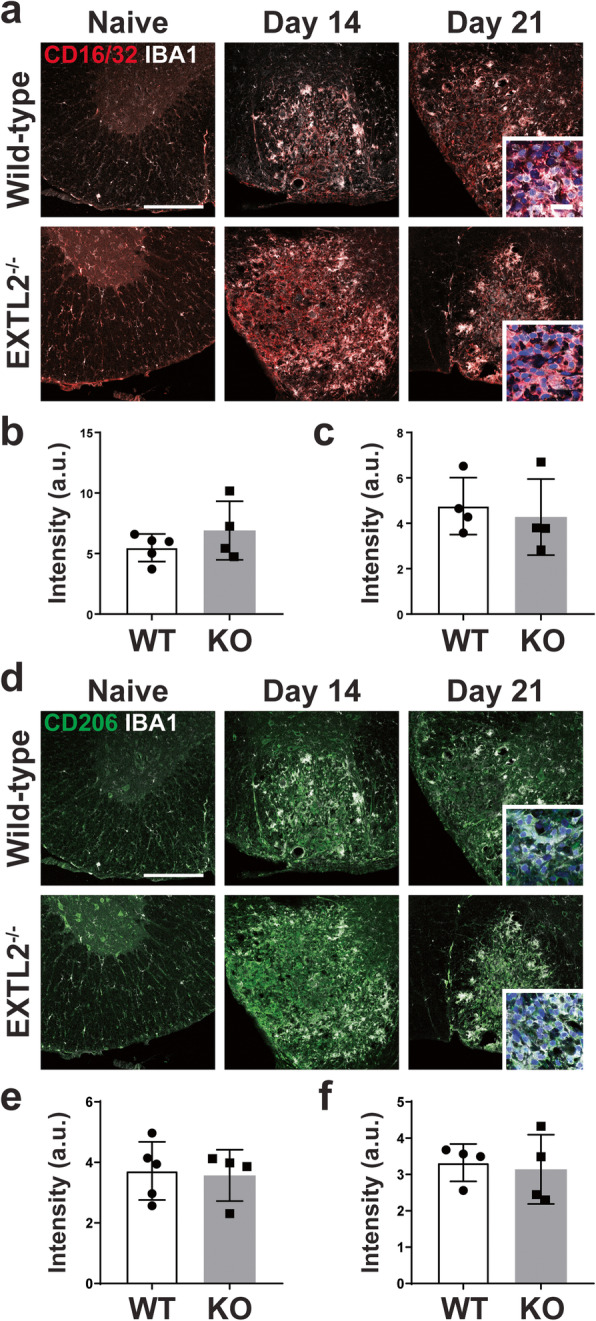
Immunolabeling of microglia/macrophages and surface markers CD16/32 and CD206. Tissue sections were probed for both CD16/32 and CD206, and representative images are shown in **a** for CD16/32 and Iba1, and **d** for CD206 and Iba1. Scale bar = 200 μm. Insets in third image are 63X objective lens magnified images of lesional Iba1^+^ (white) cells merged with either CD16/32 (red) or CD206 (green) and nuclear yellow (blue). Scale bar in inset = 20 μm. **b** Average lesion intensity of CD16/32 in lesions from 14 dpl. **c** Average lesion intensity of CD16/32 in lesions from 21 dpl. **e**, **f**)Average lesion intensity for CD206 in day 14 and day 21 lesions, respectively. Graphs show mean with standard deviation. Two-tailed, unpaired Student’s *t* test

To further resolve whether differences in polarization exist in the Iba1^+^ cells in the wild-type and EXTL2^−/−^ lesions, we probed tissue sections using immunofluorescence staining for M1-like pro-inflammatory and M2-like anti-inflammatory surface markers, CD16/32 and CD206 [33], respectively (Fig. 5a, d); the pro-inflammatory enzyme inducible nitric oxide synthase (iNOS) was attempted but did not result in reliable staining. For both CD16/32 and CD206, intensity was not significantly different between wild-type and EXTL2^−/−^ lesions (Fig. 5b, c, e, f), indicating that expression levels of these receptors are similar and that there was no apparent alterations in microglia/macrophage phenotype in EXTL2^−/−^ animals.

### Cultured EXTL2^−/−^ BMDMs upregulate iNOS, secrete more TNFα, and differentially regulate MMPs in response to CSPGs

Due to limitations of assessing the activity of Iba1^+^ cells in the lesion using fixed tissue sections, we cultured murine-derived BMDMs to examine functional or phenotypic differences between wild-type and EXTL2^−/−^ macrophages upon stimulation. As monocyte-derived macrophages constitute a significant portion of immune cells present in the lysolecithin-induced lesion (unpublished observations), we reasoned that BMDMs are an appropriate population of cells to study as a model for macrophages in the lesion. We hypothesized that excessive deposition and persistence of CSPGs in EXTL2^−/−^ animals underlies the prolonged myelin disruption and macrophage/microglia accumulation or activation, which consequently contributes to exacerbated injury. BMDMs were harvested from both wild-type C56Bl/6 and EXTL2^−/−^ mice and stimulated with a commercial CSPG mixture containing versican for 24 h. Basally, wild-type and EXTL2^−/−^ BMDMs were comparable with regard to surface expression of CD45, F4/80, CD11b, and CD68. Both groups of naïve BMDMs also exhibited similarly low expression of CD206, MHC II, and CD11c (data not shown).

**Fig. 6:**
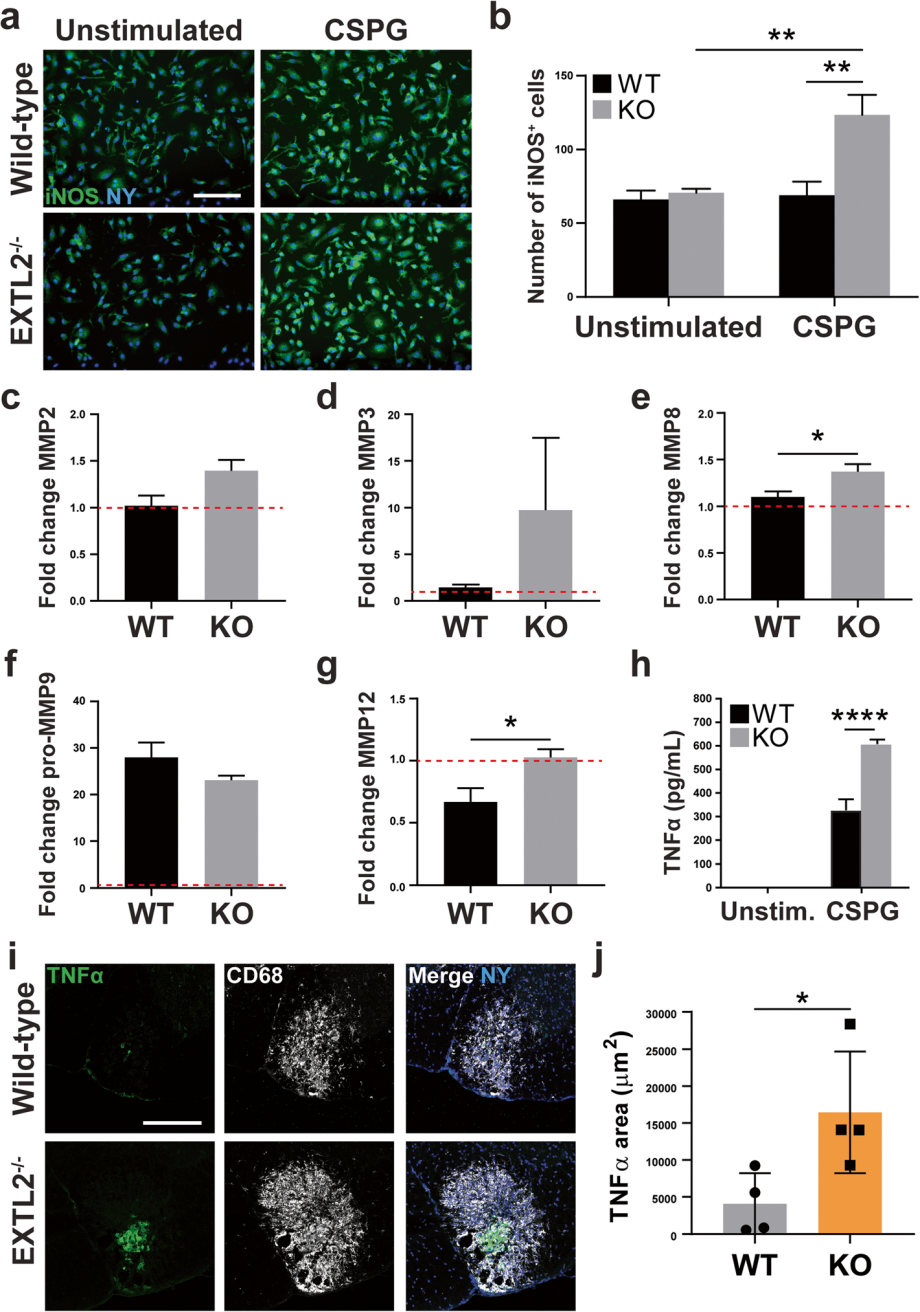
Assessment of iNOS expression in cultured BMDMs following CSPG stimulation; MMP 5-plex assay and ELISA analyses of conditioned media from cultured BMDMs. Cultured BMDMs from wild-type and EXTL2^−/−^ mice were stained for iNOS and nuclear yellow (NY). Representative images for unstimulated and CSPG-stimulated cells are shown in **a**. Scale bar = 100 μm. **b** Comparison of number of iNOS^+^ cells between wild-type and EXTL2^−/−^ cells, unstimulated or with CSPG stimulation. *n* = 4 wells per condition. Two-way ANOVA with Sidak’s multiple comparisons test. **c-g** Fold change of MMP2, MMP8, MMP3, pro-MMP9, and MMP12 detected in conditioned media from both wild-type and EXTL2^−/−^ BMDMs, unstimulated and CSPG stimulated, respectively. Ordinary one-way ANOVA with Tukey’s multiple comparisons test. Raw quantities of MMPs in pg/mL ranged from 37.1 to 58.1 for MMP2, 0.1 to 10.3 for MMP3, 305.7 to 490.0 for MMP8, 157.6 to 6265.0 for pro-MMP9, and 3936.5 to 11,558.3 for MMP12. Fold change is calculated over unstimulated cells. **h** TNFα levels in BMDM conditioned media as determined by ELISA. Two-way ANOVA with Sidak’s multiple comparisons test. **i** Representative images of TNFα and CD68 staining in day 14 lysolecithin-injured spinal cord tissue. Scale bar = 200 μm. **j** Analysis of absolute TNFα immunoreactive area within lesions at day 14 in wild-type and EXTL2^−/−^ spinal cords. All graphs are mean with SEM, except for panel **j** (mean with standard deviation). Two-tailed, unpaired Student’s *t* test. *p < 0.05, **p < 0.01

Cultured BMDMs were stained for the pro-inflammatory marker iNOS (Fig. 6a), and number of cells with enhanced iNOS expression was quantified. While similar numbers of unstimulated wild-type and EXTL2^−/−^ BMDMs expressed iNOS, CSPG stimulation resulted in a marked increase in iNOS^+^ EXTL2^−/−^ BMDMs (Fig. 6b).

Multiplex analysis to detect the presence of secreted MMP members was performed on supernatant harvested from stimulated wild-type and EXTL2^−/−^ BMDMs (Fig. 6c–g). We found that EXTL2^−/−^ BMDMs secreted more MMP8 relative to wild-type BMDMs (Fig. 6e). We observed a similar trend in MMP2 secretion; however, this did not reach statistical significance (Fig. 6c). No significant differences in MMP3 secretion were detected in any group (Fig. 6d). Upon stimulation with CSPGs, pro-MMP9 was the only MMP to be significantly increased in the wild-type BMDM conditioned medium (Fig. 6f). Unexpectedly, wild-type BMDMs decreased secretion of MMP12 in response to CSPG stimulation, and EXTL2^−/−^ BMDMs exhibited a baseline decrease in MMP12 production and did not alter MMP12 production with stimulation (Fig. 6g).

Assessment of the BMDM conditioned media using enzyme-linked immunosorbent assay (ELISA) analysis revealed significantly increased amounts of TNFα in the supernatant of EXTL2^−/−^ BMDMs compared to wild-type BMDM supernatant in response to CSPG stimulation (Fig. 6h). To corroborate this in vitro finding, we stained tissue from wild-type and EXTL2^−/−^ animals 14 days post-injury for TNFα (Fig. 6i). We found greater areas of immunoreactivity of TNFα in EXTL2^−/−^ spinal cords than wild-type (Fig. 6j), and pattern of staining appears to correlate closely with CD68, a surface marker highly expressed by microglia/macrophages [34].

### Conditioned media from EXTL2^−/−^ BMDMs do not kill cultured neurons or OPCs

**Fig. 7:**
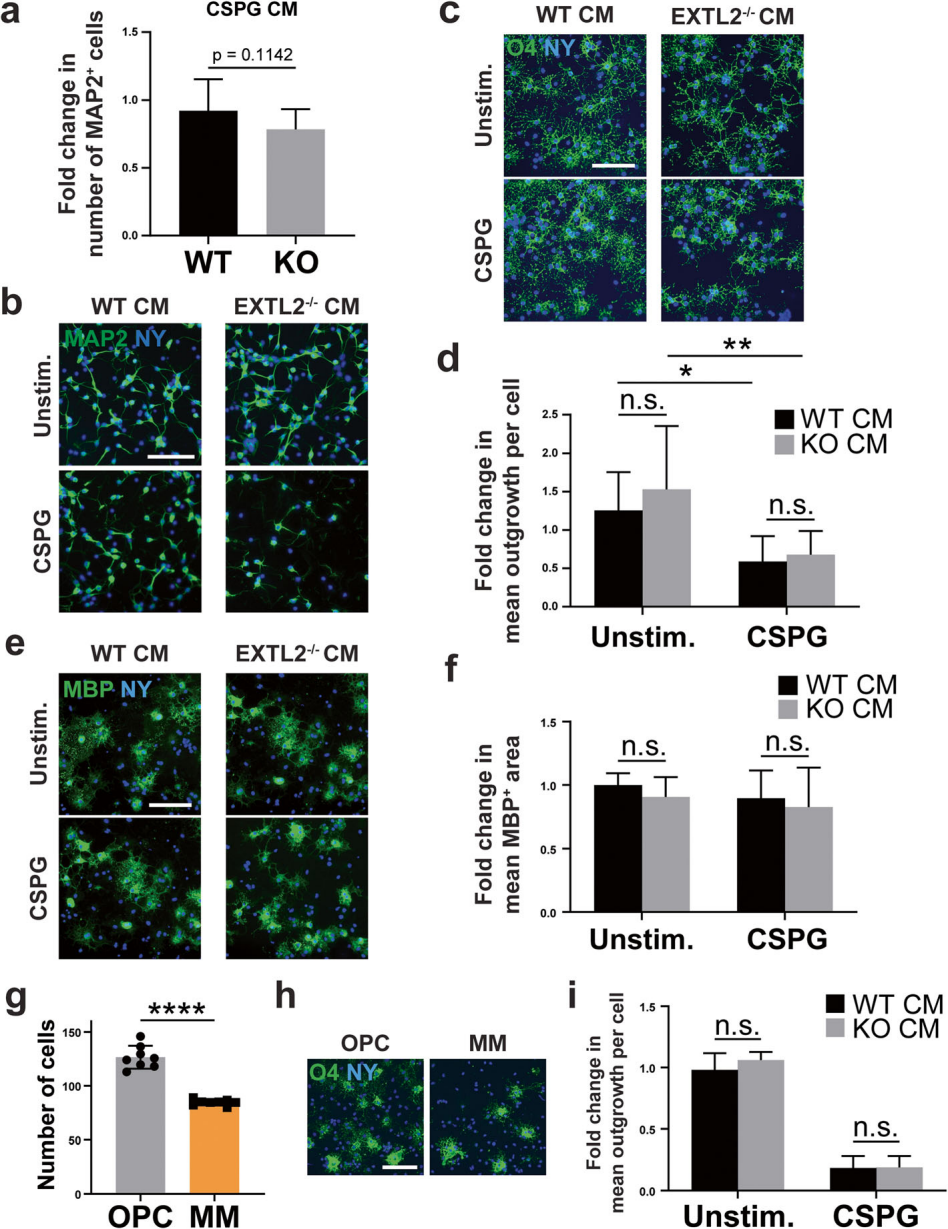
Effect of BMDM conditioned media on cultured neurons and OPCs. **a** Fold change in number of MAP2+ cells assessed for cultured neurons 24 h following application of CSPG-stimulated BMDM conditioned media (CM) (data pooled from 3 independent experiments of quadruplicate cultures each). Unpaired, two-way Student’s *t* test. **b** Images of neurons labeled with MAP2 and nuclear yellow; scale bar = 100 μm. **c** Representative images of wild-type (WT) OPCs labeled with O4 and nuclear yellow 24 h following treatment with BMDM conditioned media. Scale bar = 200 μm. **d** Comparison of wild-type and EXTL2^−/−^ BMDM conditioned media on OPC outgrowth. Fold change represents data pooled from 3 independent experiments. Two-way ANOVA with Tukey’s multiple comparisons test. n.s. = not significant. *p < 0.05. **e** Representative images of MBP^+^ oligodendrocytes 72 h following seeding into a 96-well plate with BMDM conditioned media. Scale bar = 100 μm. **f** Comparison of wild-type and EXTL2^−/−^ BMDM conditioned media on OPC differentiation 72 h after seeding. Two-way ANOVA with Sidak’s multiple comparisons test. n.s. = not significant. **g** Comparison of number of OPCs seeded either in the standard OPC media (as described in the methods section) or minimal media (MM) of DMEM containing only GlutaMAX™, sodium pyruvate, and penicillin/streptomycin. **h** Representative images of O4-stained OPCs 24 h post-seeding in either standard OPC media or MM. Scale bar = 100 μm. **i** Comparison of OPC process outgrowth 24 h post-seeding in MM, treated with conditioned media from unstimulated or CSPG-stimulated BMDMs. All graphs are mean with SEM. Two-way ANOVA with Sidak’s multiple comparisons test. n.s. = not significant

Given that in the presence of CSPGs, EXTL2^−/−^ BMDMS exhibited a more pro-inflammatory phenotype in culture, we next asked whether the pro-inflammatory nature of these macrophages may underlie the exacerbated lesion size and axonal loss observed in EXTL2^−/−^ animals following lysolecithin injection (Fig. 3). To exclude the possible contribution of contact-mediated cell death, we harvested conditioned media from cultured wild-type and EXTL2^−/−^ BMDMs stimulated with CSPGs. These conditioned media were applied to neurons in a 1:1 ratio with neuron culture media for 24 h. Although some cultures showed attrition of neuronal numbers qualitatively when exposed to the conditioned media of EXTL2^−^/^−^ BMDM, analyses of pooled results across 3 experiments (quadruplicate cultures each) did not show a quantitative loss of neurons when compared to cultures exposed to conditioned media from wild-type BMDMs (Fig. 7a, b).

Next, conditioned media were applied to primary OPC cultures. Cells were cultured for 24 h before fixing and assessment of process outgrowth using the oligodendrocyte lineage marker O4 (Fig. 7c). EXTL2^−/−^ and wild-type BMDM conditioned media had the same effect on OPC growth (Fig. 7c, d). The conditioned media of both wild-type and EXTL2^−/−^ BMDMs stimulated with CSPGs impaired outgrowth, likely due to the CSPGs contained within, but they did so to the same extent (Fig. 7d).

Three days following the application of BMDM conditioned media to OPCs, cells were assessed for MBP expression as a determinant of viability and maturation. Between wild-type and EXTL2^−/−^ conditions, the total number of cells was highly comparable, and the majority were viable and MBP-expressing (Fig. 7e). Measurement of the mean intensity of MBP staining per cell as well as the mean area of MBP staining per cell did not reveal obvious differences in both intensity (not shown) and area of MBP staining (Fig. 7f) in cultures exposed to conditioned media from wild-type or EXTL2^−/−^ BMDMs.

Finally, the presence of several growth factors normally added to the growth medium of OPC cultures may obscure differences found in the injury milieu in vivo. We therefore conducted the same 24-h OPC experiment as shown in Fig. 7 c and d, but without fetal bovine serum and growth factors added to the standard OPC growth medium. In this minimal medium (MM), OPC growth was poor in comparison to standard OPC medium (Fig. 7g, h). OPCs grown in MM were more sensitive to inhibition of process outgrowth when exposed to CSPG-stimulated BMDM conditioned media; however, there were no differences in OPC growth when exposed to wild-type and EXTL2^−/−^ conditioned media (Fig. 7i).

Overall, the OPC results in tissue culture support the in vivo observations that repopulation of lysolecithin lesions by OPCs and oligodendrocytes was not impaired in EXTL2^−/−^ mice. In contrast, using neurons in culture, we were unable to mimic the conditions in vivo whereby an exacerbated axonal loss occurred in EXTL2^−/−^-demyelinated lesions.

## Discussion

**Fig. 8:**
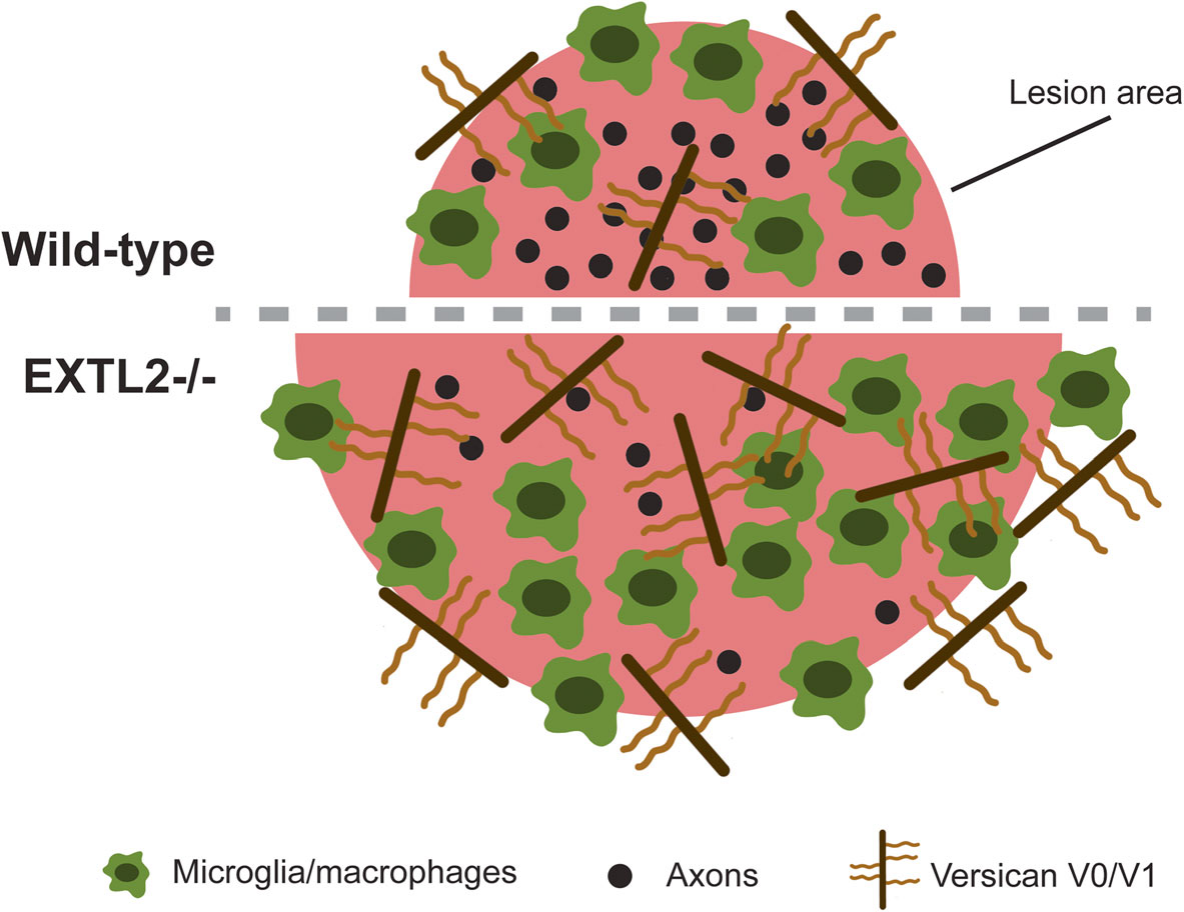
Schematic showing the phenotypes observed in EXTL2^−/−^ mice with lysolecithin-induced lesions. EXTL2^−/−^ animals have greater versican deposition into the lesion following demyelination, and this is correlated to a larger area of damage and exacerbated axon dropout within the lesion. In addition, greater amounts of Iba1 immunoreactivity was seen in EXTL2^−/−^ lesions, indicative of a more inflammatory milieu compared to that of wild-type animals

In the present study, we show that dysregulation of CSPG production following CNS injury profoundly impacts the inflammatory state of a demyelinated lesion. Loss of the enzyme EXTL2, a regulatory mechanism serving to limit CSPG synthesis, resulted in more extensive versican deposition into a lysolecithin-induced demyelinated injury, a larger area of myelin disruption with an associated decrease in axonal density within the lesion, and greater accumulation of macrophages and microglia (schematic shown in Fig. 8). The increased versican load and microglia/macrophage representation within the demyelinated lesion of EXTL2^−/−^ mice may contribute to the expansion of lesion and axonal loss, but we were not able to show in culture that macrophages from EXTL2^−/−^ mice exposed to CSPGs promote neuronal or OPC death. While our results support the increasing recognition that an elevated load of CSPGs can be pro-inflammatory and injurious in vivo, in addition to their known roles in inhibiting axonal regeneration and remyelination, the precise mechanisms by which EXTL2 deficiency promotes injury remain elusive.

Results from genetic studies strongly implicate genes from the region containing the EXTL2 gene but cannot define with precision which of the genes from the region is relevant. To date, there are no well-powered studies of MS progression so it is not possible to infer genetically whether EXTL2 (or any other gene) might be involved in the neurodegenerative aspects of the disease. In a small study of 147 patients, Saigoh et al. (2016) analyzed SNPs in the region of the related gene chondroitin sulfate β-1,4-*N*-acetylgalactosaminyltransferase-1 (ChGn-1), an enzyme also involved in CSPG synthesis, and suggested that the coding SNP (cSNP: rs140161612) might influence progression, particularly in male patients. In short, in the absence of well-powered GWAS for MS progression, it is challenging to assess genetically whether genes that regulate CSPG metabolism might be involved in MS progression.

To our knowledge, this is the first study to explore the role of EXTL2 within the CNS. Several questions remain unanswered. While we observed greater CSPG production and deposition into the lesion, the cellular sources of these CSPGs have yet to be confirmed. Multiple cell types are known to produce CSPG members, including astrocytes, OPCs, and microglia/macrophages. However, for versican V1, its major source is cells of myeloid origin [35]. In previous work, we found that CSPGs in lysolecithin lesions were expressed within Iba1+ microglia/macrophages [22]; versican V1 is the lectican CSPG member elevated within these lesions [21]. Moreover, by in situ hybridization, versican V1 transcript was detected in CD45+ cells (mostly microglia/macrophages) within lesions [18]. Whether expression within the lysolecithin lesions could be contributed by circulating versican is unknown. Another unanswered question is whether the enzymatic activity of EXTL2 is altered in the lysolecithin lesion, as this should provide further insight into the regulation of CSPG synthesis following injury.

Others have shown that inhibition of CSPG signaling through perturbation of the CSPG receptor PTPσ skews the microglia/macrophage response towards an anti-inflammatory “M2-like” state with associated increases in IL-10 [25]. Given that CSPGs, particularly versican, have a known role in the modulation of inflammation [19, 36], whether CSPGs deposited by microglia/macrophages locally can act in an autocrine or paracrine fashion to further promote inflammation while reducing “M2-like” condition is another unanswered question. In our study, we found no difference in the expression of CD16/32 and CD206, presumed “M1-like” and “M2-like,” respectively, in lesional macrophages/microglia (Fig. 5). However, previous work has suggested that a population of mononuclear phagocytes co-expresses the “M1-like” iNOS and the “M2-like” Arginase-1 simultaneously [37]. Thus, the use of only two markers in our study does not adequately reflect differences in macrophage/microglia phenotype, and cannot exclude the possibility that further analysis using a more extensive panel of markers or employing techniques such as RNA sequencing may reveal more subtle changes induced the EXTL2^−/−^ mice.

The *Extl2* gene lies close to the *Csf-1* gene on chromosome 11q11-12, so there is a possibility of mutating the *Csf-1* gene as a result of the *Extl2* manipulation. A loss of function mutation in the *Csf-1* gene in mice produces the op/op model for osteopetrosis, which is accompanied by a number of overt manifestations such as a lack of teeth, stunted growth, a domed skull, and distinct reduction in the number of macrophages across several tissues. Our EXTL2^−/−^ animals have similar abundance and distribution of Iba1^+^ cells in the spinal cord, and do not exhibit the distinctive lack of teeth. As we have not observed these outward presentations, we concluded that deleterious alterations to the *Csf-1* gene are unlikely in our *Extl2*-deficient animals. We attempted to measure CSF-1 production by BMDM from wild-type or EXTL2^−/−^ animals but were unsuccessful in detecting its expression using ELISA.

Unexpectedly, we did not find any deficiency in the extent of OPC repopulation of the lesion in the EXTL2^−/−^ animals compared to wild-type, in contrast to several lines of evidence supporting the inhibitory nature of CSPGs to remyelination. A recent study by the Yang group provides evidence that the CSPG-PTPσ signaling pathway is a suppressor of OPC migration and maturation. The authors report that perturbation of CSPG signaling (by using the PTPσ inhibitor intracellular sigma peptide) significantly accelerated recovery from dorsally injected lysolecithin and decreased severity of experimental autoimmune encephalomyelitis (EAE) [38]. It is important to note that EXTL2 is not specific to versican synthesis, and that versican was the focus of our current work because of its pronounced upregulation following injury in the lesion after lysolecithin demyelination, whereas other lectican CSPG members were not [21]. NG2, a non-lectican member of the CSPG family, is highly expressed by OPCs and upregulated upon injury. There is evidence to suggest that NG2 plays a key role in the injury response of OPCs, where loss of NG2 in OPCs reduces their mitotic index and delays remyelination [39]. Loss of EXTL2 in OPCs may similarly affect NG2 expression, thus counter-balancing the inhibitory effects of lesional versican.

Although the formation of the glial scar, largely contributed to by CSPGs, is thought to be protective in limiting the area of injury, we found a significantly greater lesional area in the EXTL2^−/−^ mice. Previous work from others has shown that the disruption of CSPG deposition in the acute phase of injury (1–3 dpl) exacerbates injury and impedes remyelination and resolution of inflammation [40]. The time points we selected for our current work do not necessarily reflect the acute injury phase, and future studies may focus on early time points to determine whether disruption of glial scar formation occurs in EXTL2^−/−^ mice. However, a report of CSPG members in active MS lesions suggests a less clear role of CSPGs. In chronic active lesions, CSPGs are seen at the borders of the lesion; however, foamy macrophages were present in the lesion core with punctate staining of CSPGs associated with the cells [4]. CSPGs, at an earlier time, may have been present uniformly throughout the lesion and are being degraded and endocytosed by macrophages from the lesion center. This, supported by our previous study in EAE [18], implies that CSPGs may be exacerbating inflammation rather than serving a neuroprotective role.

This study did not address the closely related group of ECM to the CSPGs, the heparan sulfate proteoglycans (HSPGs). Due to the N-acetylglucosaminyltransferase activity of EXTL2, it has been proposed that EXTL2 also plays a role in HSPG synthesis, where it is thought to be a promoter of HSPG production rather than a regulator. There is some evidence to suggest that knockdown of EXTL2 may reduce HSPG synthesis [41, 42]. HSPGs are largely regarded as a growth-permissive ECM member. Whether HSPGs are downregulated as a result of EXTL2 knockout was not determined, and consequently, what impact reduced HSPG production may have on inflammation and repair.

Our work and others have shown that approaches to reducing CSPGs may be key in promoting regeneration in the CNS. The bacteria-derived enzyme chondroitinase ABC has previously been used in SCI and white matter ischemia models, where treated animals exhibited histological and functional improvements over untreated animals [14, 24, 27]. Pharmacological agents that reduce biosynthesis of CSPGs have been proposed as an alternative to CSPG-cleaving enzymes [21, 22]. Protamine is a heparan neutralizing drug that was also found to block CSPG-mediated inhibition on OPC growth in culture [43].

Overall, our data supports previous reports that EXTL2 is a negative regulator of CSPG biosynthesis. We have shown that the loss of EXTL2 results in the excessive production of versican in response to demyelination, which consequently results in protracted response from microglia/macrophages. This study provides the first evidence for a role of EXTL2 in regulating the responses to CNS demyelination and identifies a novel target for future studies investigating CSPGs and their role in CNS injury. Our results also set the stage to evaluate the mechanisms by which the CSPG-elevated microenvironment leads to exacerbation of lesion size following demyelination.

## Conclusions

This study suggests a potential genetic MS risk association with the *Extl2* gene, which encodes for EXTL2, a negative regulator of CSPG biosynthesis. We conclude from the present study that CSPGs may be a potent modulator of neuroinflammation in the context of a demyelinating injury. Dysregulation of the EXTL2 enzyme results in increased accumulation of CSPGs within the white matter injury also associated with a decreased axonal density and exacerbated injury area. We highlight the accumulation of CSPGs as a promoter of axonal injury, through the potential augmentation of the inflammatory macrophage response. In vitro cell culture results suggest that CSPGs are able to promote an inflammatory response through induction of TNFα secretion, as well as some MMPs. In addition to their better described inhibitory roles for remyelination and axonal repair in the CNS, these results indicate that CSPGs are also promoters of detrimental neuroinflammation in white matter demyelination.

## Data Availability

All data generated or analyzed during this study are included in this published article.

## Abbreviations

BMDM: Bone marrow-derived macrophages
ChABC: Chondroitinase ABC
CSPGs: Chondroitin sulfate proteoglycans
ECM: Extracellular matrix
EXTL2: Exostosin-like 2
HSPGs: Heparan sulfate proteoglycans
MBP: Myelin basic protein
MMPs: Matrix metalloproteinases
NF200: 200 kDa neurofilament
OPC: Oligodendrocyte precursor cell
PBS: Phosphate-buffered saline
PFA: Paraformaldehyde
SNP: Single nucleotide polymorphism
TNF-α: Tumor necrosis factor α
